# Expression of the miR-302/367 microRNA cluster is regulated by a conserved long non-coding host-gene

**DOI:** 10.1038/s41598-021-89080-z

**Published:** 2021-05-27

**Authors:** Karim Rahimi, Annette Christine Füchtbauer, Fardin Fathi, Seyed Javad Mowla, Ernst-Martin Füchtbauer

**Affiliations:** 1 Department of Molecular Biology and Genetics, Aarhus University, C.F. Møllers Alle 3, 8000 Aarhus C, Denmark; 2 Cellular and Molecular Research Center, Research Institute for Health Development, Kurdistan University of Medical Sciences, Sanandaj, Iran; 3 Molecular Genetics Department, Faculty of Biological Sciences, Tarbiat Modares University, Tehran, Iran

**Keywords:** Biological techniques, Cell biology, Developmental biology, Genetics, Molecular biology, Stem cells

## Abstract

MicroRNAs are important regulators of cellular functions. *MiR-302/367* is a polycistronic miRNA cluster that can induce and maintain pluripotency. Here we investigate the transcriptional control and the processing of the miR-302 host-gene in mice. Our results indicate that the mmu-miR-302 host-gene is alternatively spliced, polyadenylated and exported from the nucleus. The regulatory sequences extend at least 2 kb upstream of the transcription start site and contain several conserved binding sites for both transcriptional activators and repressors. The gene structure and regulatory elements are highly conserved between mouse and human. So far, regulating *miR-302* expression is the only known function of the miR-302 host-gene. Even though we here only provide one example, regulation of microRNA transcription might be a so far little recognized function of long non-coding RNA genes.

## Introduction

MicroRNAs (miRNAs) are short non-coding RNAs regulating gene activity at a post-transcriptional level. MiRNAs localized intergenicly are transcribed by RNA polymerase II or III (pol-II or pol-III), while intragenic miRNAs are co-transcribed with their coding or non-coding host-genes by RNA pol-II^1–3^. For achieving tissue-specific transcription, RNA pol-III is not suitable while RNA pol-II can perform complex regulated transcription.

The human and murine *miR-302/367* (here termed *miR-302*) clusters are both located in intron 8 of the LA related protein 7 (*LARP7/Larp7*) gene in antisense direction and encode four stem cell specific miRNAs including *miR-302b/c/a/d* and *miR-367*, transcribed from a pol-II promoter and generate from the same primary transcript. *MiR-302a-d* are highly related and share the same seed sequence, which is also shared with *miR-290–295* in mice and *miR-373* in humans. The co-transcribed *miR-367* has a different seed sequence^4^. The target genes of *miR-367* include *Smad7* and the downstream TGF-ß signaling in cells. It promotes invasion and metastasis of human pancreatic cancer cells^5^. Also, *miR-367* causes proliferation and stem cell-like behavior in medulloblastoma cells^6^. It has been shown that *miR-302* has a significant function in cell reprograming e.g. initiating genomic DNA demethylation, activating ESC-specific transcription factors, blocking developmental and differentiating pathways and preventing stem cell tumorigenicity^7^.

In humans, the hESC-specific expression of the hsa-miR-302 host-gene is ascribed to 525 bp immediately upstream of the transcription start site^8,9^. *MiR-302* represses differentiation of stem cells and supports somatic cell reprogramming in a variety of mammals^7,10,11^ independent of the addition of pluripotency transcription factors like OCT4, SOX2, KLF4, and MYC^12,13^. Interestingly, all these transcription factors, bind to the promoter region of the miR-302 host-gene in both mice and humans^14^ and the expression level of *miR-302* correlates with the expression level of Oct4^10^. Furthermore, the *miR-302* promoter is a direct target of canonical WNT signaling, which depends on the OCT4/NANOG binding sites^15^, and *miR-302*s repress translation of the transcription factor COUP-TFII, a transcriptional repressor of *Oct4* and *Sox2*^16^. *MiR-302*s thus act as positive regulators of OCT4^17^. *Hsa-miR-302*s have also been shown to be highly correlated with the expression pattern of cancer stem cell markers^18^. The functional independence of *miR-302* is further emphasized by the fact that it can regulate tumorigenicity by suppressing both cyclin E-CDK2 and cyclin D-CDK4/6 during G1-S cell cycle transition^19,20^. The function of the *miR-302* cluster and potential therapeutic applications have been reviewed elsewhere^21–23^.

During murine embryonic development, expression of the *mmu-miR-302* cluster is rapidly down regulated after day 8 of gestation if assayed by whole embryo analysis^4^, but little is known about expression in individual stem cells. RT-PCR and whole mount in situ hybridization showed *mmu-miR-302* expression in the developing lung of murine embryos up to day 15 of gestation^14^.

In mice and humans, *pri-miR-302* is generated by splicing of an intron which is part of a long non-coding host-gene that so far has not been assigned any additional function (mmu-miR-302 host-gene LOC110008574 (Gm51018) and hsa-miR-302 host-gene LOC109864269). If *miR-302* followed faithfully the expression of *Oct4* as indicated by^10^, the *miR-302* cluster might be simply embedded in an intron of *Oct4* or one of its other transcription factors i.e. *Sox2* or *Nanog*. We therefore asked whether the transcriptional regulation of the miR-302 host-gene is more complex and requires more complex regulatory sequences and RNA processing such as splicing and polyadenylation.

Since *miR-302* is a stem cell specific microRNA^24^, we studied and analyzed the expression and transcript structure of the miR-302 host RNA in murine embryonic stem (ES) cells, F9 embryonic carcinoma (EC) cells and cancer stem cell like cells which we obtained from primary culture of teratoma cells by selection for miR-302 host-gene expression^25^.

Our promoter/enhancer analysis revealed a high complexity of the transcriptional regulation of the miR-302 host-gene in both mice and humans, which exceeds the regulation of its individual transcription factors. Furthermore, we noticed that the regulatory and RNA processing elements like enhancer, promoter, or splice signals, are highly conserved between human and mouse. RNA processing has an important influence on transcriptional activity and the conservation might indicate that transcriptional regulation and processing of the *miR-302* cluster is an important function of the miR-302 host-gene, for which no other function has been annotated so far.

## Materials and methods

### Bioinformatics sequences analysis

To predict the stucture of the mmu-miR-302 host-gene and its upstream regulatory sequences in comparison to the hsa-miR-302 host-gene, different software, and online bioinformatics tools were used (Table S2) including TRANSFAC, the Transcription Factors Binding Sites tool^26^.

### Statistical data analysis

Analysis of Variance (ANOVA) was used to determine whether the different expression levels among the genes and different promoter elements are statistically significant. All experiments were performed in triplicates and the results are shown as the mean ± standard deviation (SD). A value of *p < 0.05 was considered to be statistically significant, if not otherwise mentioned. The significance of the difference between the samples was analyzed by the Tukey test, as a *post hoc* test. All statistical data analysis was performed using GraphPad Prism software version 7.00 for Windows, GraphPad Software, San Diego, California USA.

### DNA and RNA preparation and PCR/qPCR condition and setup

Genomic mouse DNA was isolated as previously described^27^. Total RNA was purified using TRIzol (Invitrogen). All promoter elements were amplified using *Pfu* DNA polymerase (ThermoFisher). AmpliTaq Gold 360 Master Mix (ThermoFisher) was used for genotyping PCR and RT-PCR reactions. PCR products were visualized on 1% agarose gel electrophoresis and confirmed by Sanger sequencing.

All cDNA was synthesized from 1 μg of DNase treated total RNA using random hexamer oligonucleotides and M-MLV Reverse Transcriptase (Invitrogen) in 20 μl reaction mix. Synthesized cDNA was dilluted 20 times and 4 μl of it was used for each qPCR reaction. Platinum SYBR Green I Master kit (Thermo Fisher) was used for qPCR quantification according to the manufacture’s instruction and ran on Light cycler 480 II instrument (Roche).

In addition to Western blot, qPCR was used for validation of the cell fractionation efficiency using different cytoplasm and nucleus specific marker transcripts. Localization of miR-302 host RNA was also quantified in both cytoplasm and nucleus.

Two biological replicates were used for the analysis. *Gapdh* and *Hprt* were used as cytoplasmic specific transcript and *Malat-1* was used as nuclear specific marker. *Oct4* was used as both stem cell marker and cytoplasm specific marker. For quantification of miR-302 host RNA, an intron spaning primer pair was used to targeting both exons of the host RNA. All qPCR reactions were carried out in technical triplicates and cytoplasmic and nuclear fractions were used for targeting of each marker transcript separately. The obtained triplicate Ct were transformed (2^-Ct^) and averaged. For data visualization, a relative localization of each transcript in the two fractions was calculated. Primer sequences (Table S1) and PCR programs are described in the supplement.

### Rapid amplification of cDNA ends (RACE)

Rapid Amplification of cDNA Ends (RACE) for the 5′ and 3′ ends was performed using ExactSTART Eukaryotic mRNA 5′- & 3′-RACE Kit (Epicentre) according to the manufacture’s instruction. For 5′-RACE “mmu-miR-302 transcript rev4” (5′-GGATTTGCCTTTGTGGAA) was used as reverse and internal primer. For 3′-RACE “mmu-miR-302 transcript fwd6” (5′-AACCACATTGCCACATTTCCCA) was used as forward and internal primer. Primer sequences (Table S1) and PCR programs are described in the supplement.

### Cloning and luciferase assay

TOPO TA Cloning (Invitrogen) was used to clone PCR products. All sequencing was performed by GATC Biotech, Konstanz, Germany. Sequences were analyzed using CLC main workbench (Qiagen). Different upstream genomic regions of the mmu-miR-302 host-gene were inserted into the psiCheck2 Promega vector (GenBank Accession Number: AY535007) upstream of the *Renilla* luciferase gene replacing the SV40 promoter. This vector also encodes *Firefly* luciferase driven by the TK promoter, which was used as an internal standard to normalize for transfection efficiency. The vector with and without the SV40 promoter served as positive and negative control respectively. Notably, the vector backbone itself contains cryptic TF biding sites which result in a relatively high 'background' expression level. This gives the possibility to observe repressor activity but also reduces the signal to noise ratio. FuGene 6 (Promega) transfection reagent was used for all transient transfections. Dual-Luciferase Reporter Assay System kit (Promega) was used for luciferase assays.

### Cell culture condition

CJ7 murine ES cells^28^ were grown in ES cell medium of DMEM (Gibco 41,965–039), containing 15% Fetal Calf Serum (2602-P250915; Pan Biotech GmbH), 1000 U/ml LIF (Invitrogen PMC9484), 1% glutamine (Gibco 25,030), 1% Penicillin–Streptomycin (Gibco 15,070–063), 1% non-essential amino acids (Gibco 11,140), 1% fresh 10 mM β-mercaptoethanol and 1% 100X nucleosides mix on mitotically inactivated embryonic fibroblast cells, unless otherwise mentioned. Gelatin (Sigma-Aldrich G1393) was used for covering all the culture dishes before seeding the cells.

### ES cells electroporation

In 800 µl of complete PBS, 6.6 × 10^6^ ES cells were electroporated with 25 µg of linearized vector using 240 V, 500 μF. Cells were washed once with 10 ml ES cell medium and seeded on four six-centimeter dishes with feeder cells. After 24 to 36 h, transfected cells were selected for 6 to 8 days with neomycin at 350 µg/ml (Roche Diagnostics GmbH, 04,727,894,001, potency 785 μg/mg). Resistant clones were picked individually.

### Teratoma generation and stem cells like derivation

Teratomas were generated by subcutaneous injection of 50 µl Hank's solution containing 1600 ES cells stably transfected with a neomycin resistance gene driven by 2.1 kb upstream genomic region (part ABC) of the mmu-miR-302 host-gene (chr3:127,542,969–127,545,132 plus strand, GRCm38/mm10). Two 7 month old male 129 Sv/Pas mice were injected on both sides of the back. 129 Sv/Pas mice are almost isogenic with the 129S3/SvImJ derived ES cells and show no immune response upon engrafting of these cells^25^. Mice were kept under standard conditions with food and water supplied ad libitum, monitored daily and sacrificed by cervical dislocation after 21 days when the tumors had reached a size of approximately 1 cm^3^. Tumors were carefully dissected avoiding contamination with surrounding tissue and divided for RNA preparation, histology, and cell culture. For cell culture, samples were cut into small pieces of 3–4 mm, washed 3 times with calcium and magnesium free (CMF) PBS and immersed in 0.25% trypsin for 6 h at 4 °C. Then the excess trypsin was removed and the tissue was incubated for 30 min at 37 °C. Trypsin was inactivated with DMEM containing 10% FBS and, 10^6^ cells were seeded per well of 12 wells plate in ES cell medium without selection. After two weeks plates were confluent with differentiated cells like myoblasts and adipoblasts. No stem cell like cells were visible. At this time, G418 (300 µg/ml) was added to the medium, which efficiently removed the differentiated cells. After a few days, ES cell like cells appeared in the culture. Cells were grown under continuous G418 selection, however, in some cases, selection was stopped 4 days prior to the transfection with the luciferase reporter constructs.

### Cell fractionation

A modified version of the fractionation protocol published by Mayer and Churchman^29^ was followed for preparation of cytoplasmic and nuclear fractions. For each of the two replicates, approximately 1.5 × 10^7^ cells on a 10 cm dish were washed twice with 10 ml CMF-PBS buffer and scraped off the plate in 1 ml of CMF-PBS buffer. Samples were collected by centrifugation in an RNase-free 1.5 ml Eppendorf tube at 500 g for 2 min at 4 °C. The supernatant was completely removed and discarded. Then 200 μl of cytoplasmic lysis buffer (0.15% (v/v) NP-40, 10 mM Tris–HCl (pH 7.0), 150 mM NaCl, 20 U Ribolock RNase inhibitor (Thermo Fisher Scientific) per reaction and 5 µl of protease inhibitor cocktail mix (Roche) per reaction) were added to the cell pellet and mixed by pipetting ten times. The cell lysate was incubated on ice for 5 min. Using a 1,000-μl pipette tip, the cell lysate was carefully added onto 500 μl of sucrose buffer (10 mM Tris–HCl (pH 7.0), 150 mM NaCl, 25% Sucrose (w/v), 40 U Ribolock RNase inhibitor (Thermo Fisher Scientific) per reaction and 10 µl of protease inhibitor cocktail mix (Roche) per reaction) without mixing the two separate layers. Cell nuclei were collected at the bottom of the tube by centrifugation at 16,000 g for 10 min at 4 °C. The supernatant was collected as cytoplasmic fraction. Of this, 50 μl were mixed with 50 μl 2 × SDS loading buffer (125 mM Tris–HCl pH 6.8, 20% glycerol, 5% SDS, and 0.2 M DTT) for Western blot analysis. The remaining supernatant was used for RNA extraction following the Trizol protocol.

The pellet containing the nuclei and was washed with 800 μl nuclei wash buffer [0.1% (v/v) Triton X-100, 1 mM EDTA, 100 U Ribolock RNase inhibitor (Thermo Fisher Scientific) per reaction and 20 µl of protease inhibitor cocktail mix (Roche) per reaction and add 940.5 μl 1 × PBS per reaction] and collected by centrifugation at 1,150 g for 1 min at 4 °C, and the supernatant was discarded completely. This procedure was repeated for a total of three times. The final nuclear pellet was resuspended in 200 μl of nuclei lysis buffer [1% (v/v) NP-40, 20 mM HEPES (pH 7.5), 300 mM NaCl, 1 M Urea, 0.2 mM EDTA, 1 mM DTT, 20 U Ribolock RNase inhibitor (Thermo Fisher Scientific) per reaction and 5 µl of protease inhibitor cocktail mix (Roche) per reaction] by vortexing and incubated on ice for 2 min. A volume of 50 μl was taken for Western blot analysis, and added to 100 µl of CMF-PBS buffer and mixed with 150 μl of 2 × SDS loading buffer. The rest of the resuspended nuclear fraction was mixed with 800 μl Trizol and used for RNA extraction according to the manufacturer’s instruction.

### SDS-PAGE and Western Blot

For Western blot, 10 μl from each cell fractions were resuspended separately in an equal volume of 2 × SDS loading buffer [125 mM Tris–HCl pH 6.8, 20% glycerol, 5% SDS, and 0.2 M DTT] and briefly boiled at 95 °C for 5 min and loaded on a 12% Tris–Glycine SDS-PAGE gel and run for approximately 1.5 h at 125 V. The proteins were transferred to an Immobilon-P Transfer Membrane (EMD Millipore) by wet-blotting overnight at 4 °C and 25 V. To pre-block unspecific protein binding, the membrane was incubated for 1 h at room temperature with 10% skimmed milk and followed by 1 h incubation with primary antibody (anti-β-tubulin (1:2,000); Millipore (AB_309885), anti-histone H3 (1:20,000); Abcam (AB_302613)) and 1 h with secondary antibody (In order, anti-mouse antibody (Polyclonal, Goat) (1:5,000), Dako AB_2617137 and anti-rabbit antibody (Polyclonal, Goat) (1:5,000), Dako AB_2617138). After each antibody incubation, the membrane was rinsed 3 × for 5 min in 1 × PBS + 0.05% Tween-20 and 1 × for 5 min wash with 1 × PBS. The protein bands were developed using SuperSignal West Femto Maximum Sensitivity Substrate kit (Thermo Fisher Scientific) and Amersham Hyperfilm ECL (GE Healthcare).

### Ethics statement

All animal and experimental protocols were carried out in compliance with ARRIVE guidelines and have been performed with the approval and permission of the Danish Ministry for Food, Agriculture, and Fishery and according to the regulations of the Danish Animal Experiments Inspectorate called Dyreforsøgstilsynet (permission number: 2015–15-0201–00,517).

## Results

### Genomic structure of the mmu-miR-302 host-gene, its transcription, and processing

**Figure 1:**
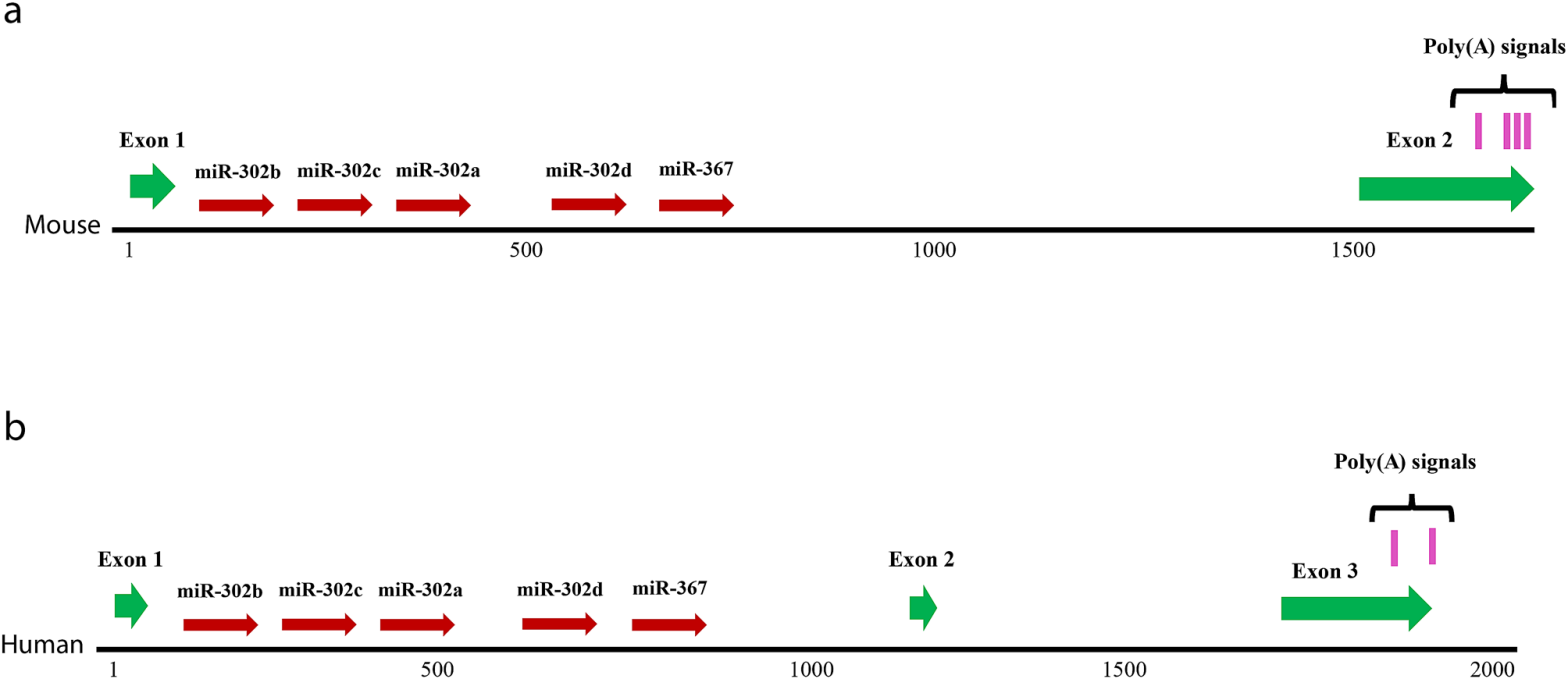
Comparison of murine (**a**) and human (**b**) miR-302 host-gene structures (LOC110008574 (Gm51018) and LOC109864269 respectively). The genomic coordinates for the mmu-miR-302 host-gene and the hsa-miR-302 host-gene are located at chr3:127,545,089–127,546,901 plus strand (GRCm38/mm10) and chr4:112,646,720–112,648,703 minus strand (GRCh38/hg38) respectively. The murine gene lacks the central exon but has four potential poly adenylation signals. Note the different scales.

Comparison of the murine and human miR-302 host-gene sequences revealed conservation of the microRNA cluster within the intron of a non-coding gene. In the mouse, none of the potential start codons is preceded by stop codons, and none of the potential small peptides can be found in protein databases. In contrast to the hsa-miR-302 host-gene^8,9^, we found no indication of an alternative exon in the murine genome. However, two splice donor sites, the first one being conserved in humans, open the possibility for alternative splicing. Similar to the hsa-miR-302 host gene, which has two p(A) signals, the mmu-miR-302 host-gene bears the possibility of alternative polyadenylation with three to four p(A) signals at the end of the second exon. Figure 1 shows a comparison of the human and murine miR-302 host-gene structure.

**Figure 2:**
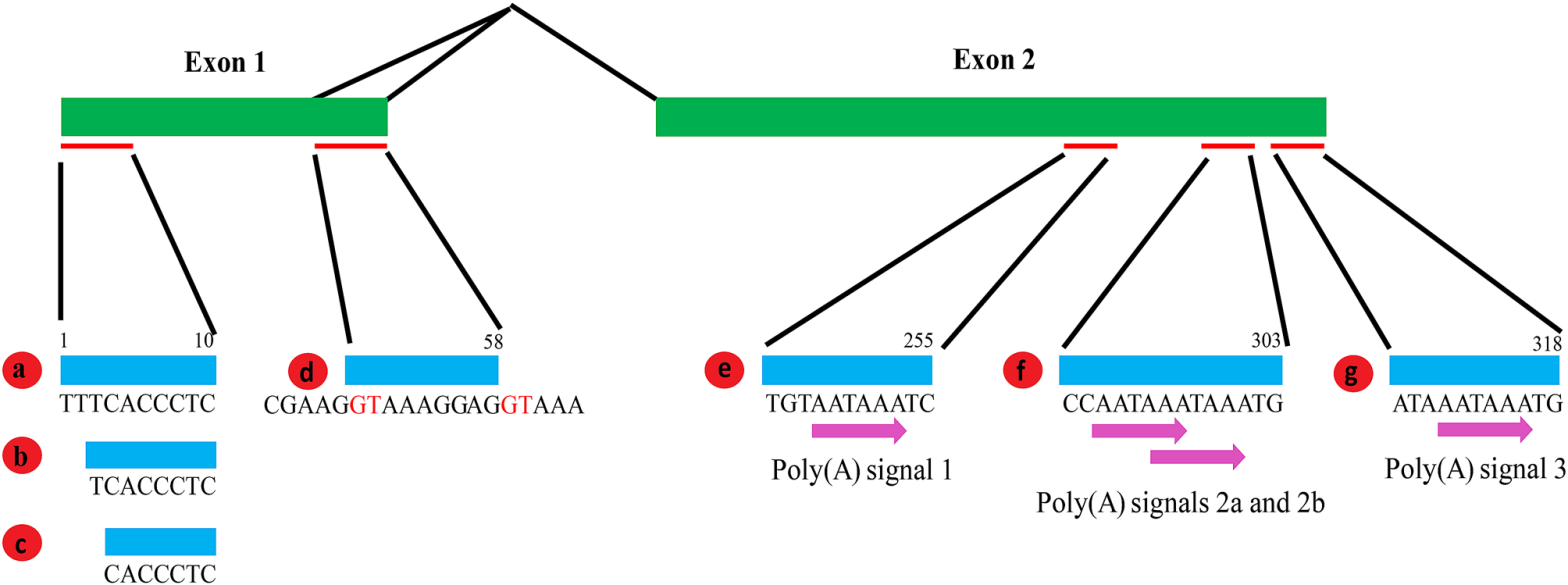
Processing of the mmu-miR-302 host transcript. The numbering is based on the longest variant of the transcript. (**a**–**c**) Transcription start sites. (**d**) Alternative splicing, splice donors shown in red. (**e**, **f**, **g**) Different 3′ terminations. The corresponding sequnces were deposited to GenBank with accession numbers: a: KT932380; b: KT932381; c: KT932382; d: KT932383; e: KT932386; f: KT932384; g: KT932385.

Performing 5′ and 3′ RACE revealed three transcription start sites (variants a, b, and c in Fig. 2) and confirmed the alternative use of the poly(A) signals for the mmu-miR-302 host-gene. All six sequences obtained from murine ES cells were polyadenylated immediately after the last poly(A) signal indicating that the overlapping poly(A) signals 2a/b were used which are located 12 and 8 base pairs upstream respectively (f in Fig. 2). Two out of six sequences obtained from murine teratomas used the same poly(A) signal, while the poly-A sequence in three out of six started 12 bases downstream of the last poly(A) signal (g in Fig. 2). In a single sequence, the first poly(A) signal was used (e in Fig. 2).

RT-PCR followed by Sanger sequencing confirmed the absence of a central exon and the alternative use of two splice donor sites (d in Fig. 2). In three out of three sequences obtained from murine ES cells, this longer version of exon 1 was found while seven out of seven sequences obtained from ES cell-derived teratomas showed splicing at the first splice donor site.

### Subcellular localization of the mmu-miR-302 spliced host RNA

**Figure 3:**
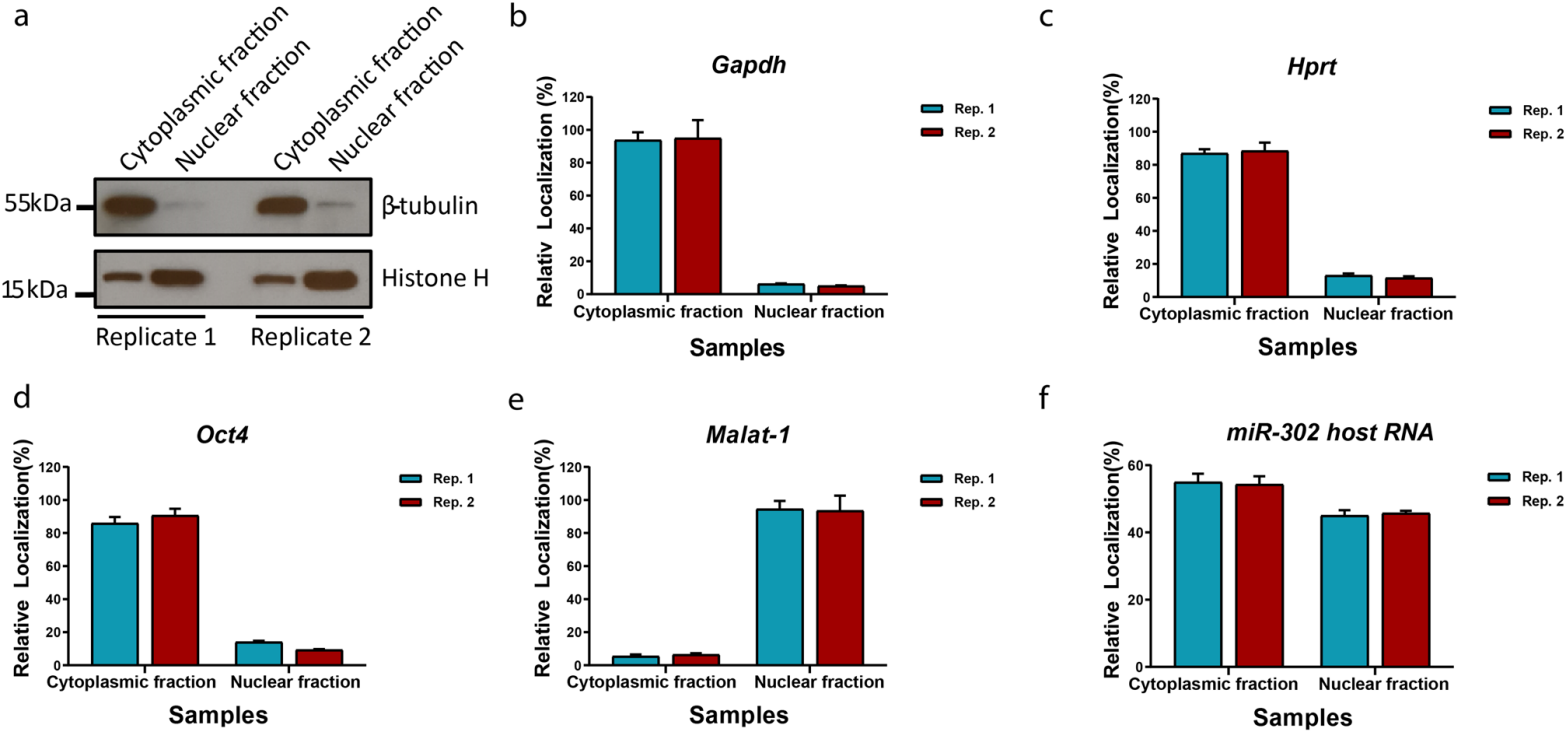
Distribution of the mmu-miR-302 host RNA in the cell. (**a**) Western blot analysis of sub cellular fractions for two biological replicates showing little β-tubuli in the nucleus and high enrichment of histone H3 in the nucleus indicating efficient separation of cytoplasmic and nuclear compartments. Equal amounts of the fractions were loaded. The original full picture of the entire Western blot membranes are shown in the suplemental Figure S2. QRT-PCR quantification of the *Gapdh* (**b**), *Hprt* (**c**) and *Oct4* (**d**) as cytoplasmic refernce RNA transcript and *Malat-1* (**e**) as nuclear reference RNA confirmed efficient fractionation. MiR-302 host RNA (**f**) found in both cytoplasmic (> 54%) and nucleus (> 45%) indicating it is significantly exported to the cytoplasm, however its accumulated level in the cytoplasm is lower and not comparable to the reference RNA transcripts. Bar plots show the average of the transformed Ct (2^-Ct^) calculated to visualize the relative localization level of each respective transcript in percentage of the cytoplasmic and nuclear fractions of the same sample. Error bars representing ± SD, n = 3 and, *p* < 0.05 as significance level.

Our RACE analysis showed that the mmu-miR-302 host RNA is capped, spliced and poly-adenylated. We therefore asked whether it might be exported to the cytoplasm. To investigate this, we performed quantitative RT-PCR from the cytoplasmic (Cyt) and nuclear (Nuc) RNA fractions. The efficiency of fractionation was validated by Western blot using antibodies against the cytoplasmic protein β-tubulin and nuclear specific protein histone H3 (Fig. 3a). Additionally, the fractionation efficiency was confirmed by qRT-PCR quantification and distribution analysis of several housekeeping RNA transcripts including *Gapdh* (Fig. 3b) and *Hprt* (Fig. 3c) as cytoplasmic RNA species. *Oct4* (Fig. 3d) was used both as cytoplasmic RNA and as stem-cell specific marker. *Malat-1* (Fig. 3e) was used as nuclear RNA control. Even though not 100% pure, the fractions were greatly enriched. Importantly, the distribution of *Malat-1* RNA proofed that the cytoplasmic fraction contained very little nuclear RNA. We found more than 50% of the miR-302 host RNA to be exported from the nucleus, which is less than what we found for the translated mRNAs, but significantly more than for the nuclear *Malat-1* RNA (Fig. 3f).

### Analysis of miR-302 upstream regulatory sequences

The conserved 500 bp region upstream of the mmu-miR-302 host-gene contains, among others, binding sites for OCT4, SOX2, and NANOG and has been described as the stem cell specific regulatory element (Supplementary Fig. S1). However, mmu-miR-302 host-gene expression is relatively low in naive murine ES cells compared to primed stem cells like murine epiblast stem cells or human ES cells^30^. This is surprising because all the major transcription factors that bind within the first 500 bp are present in all these cells. In order to test if additional regulatory elements contribute to the mmu-miR-302 host-gene expression, we analyzed 2.1 kb of the upstream genomic sequence using the TRANSFAC software^26^.

Among others, the sequence upstream of -600 bp contains additional binding sites for OCT4, SOX2, and NANOG, GATA-6, DMRT4, REX1, FOXA2 (HNF-3beta), FOXC1 and TRP53, many of which are conserved between mouse and human (Fig. S1).

**Figure 4:**
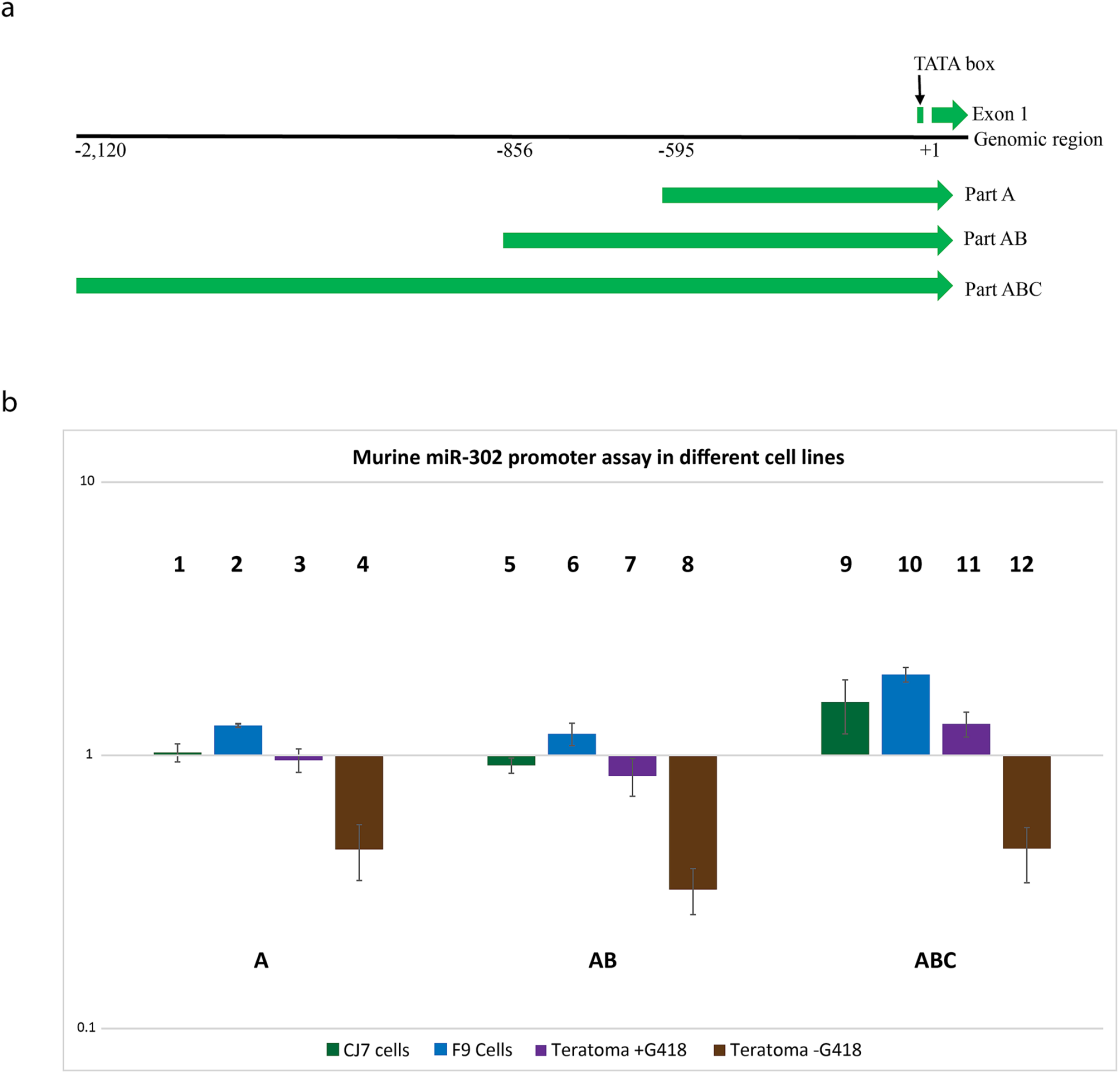
Functional analysis of the 2.1 kb upstream genomic region of the mmu-miR-302 host-gene. (a) Schematic representation of the 3 regions analyzed: part A (+ 45 to -595), part AB (+ 45 to −856), and part ABC (+ 45 to −2120). (**b**) Comparison of the promoter/enhancer activity of the three regions in different cell types, normalized to the expression level of the promoter-less reporter. The baseline expression level is relatively high, which allows to test for repressor activity, which appears as values below 1. F9 cells (2, 6, 10) show consistently a higher reporter expression than ES cells (1, 5, 9) or teratoma derived stem cells selected for miR-302 host-gene expression (teratoma + G418: 3,7,11). If selection is stopped, teratoma derived cells start to differentiate and lose reporter expression (teratoma -G418: 4, 8, 12). Bars represent mean ± SD, n = 3. Statistical comparison of data: 1 vs. 4 *p* < 0.01, 2 vs. 4 *p* < 0.0001, 3 vs. 4 *p* < 0.01, 5 vs. 8 *p* < 0.01, 6 vs. 8 *p* < 0.0001, 7 vs. 8 *p* < 0.01, 9 vs. 10 *p* < 0.05, 9 vs. 12 *p* < 0.0001, 10 vs. 11 *p* < 0.001, 10 vs. 12 *p* < 0.0001 and 11 vs. 12 *p* < 0.0001, 1 vs. 9 *p* < 0.05, 5 vs. 9 *p* < 0.01, 2 vs. 10 *p* < 0.01 and 6 vs. 10 *p* < 0.001. All other comparisons are not significant (*p* > 0.05). Note the logarithmic scale.

To analyze the contribution of the upstream genomic sequences on mmu-miR-302 host-gene expression, the 2.1 kb region of the mmu-miR-302 host-gene upstream regulatory sequence was divided into three areas which to some degree represent conserved clusters of TF binding sites (Fig. 4a). Luciferase-based reporter vectors containing the different upstream regulatory regions A (+ 45 to -595—chr3:127,544,494–127,545,132 plus strand, GRCm38/mm10), AB (+ 45 to -856—chr3:127,544,233–127,545,132 plus strand, GRCm38/mm10) and ABC (+ 45 to -2,120—chr3:127,542,969–127,545,132 plus strand, GRCm38/mm10) were designed. The vector containing the SV40 promoter upstream of the Renilla luciferase gene and the promoter-less vector were used as positive and negative control respectively. All data were normalized to the relatively high baseline expression from the promoter-less vector. Therefore values below 1 represent transcriptional repression. We compared the transcriptional activity of these five reporter constructs in ES cells, F9 EC cells and teratoma derived cancer stem cell like cells which were isolated using neomycin resistance driven by the ABC upstream region. These teratoma derived cells are *bona fide* cancer stem cells which quickly lose their stem cell characteristics when the selection pressure is relived^25^. We include these cells due to the cancer relevance of *miR-302* and because it gave us the possibility to compare basically the same cells in an undifferentiated and differentiated state.

Only the full-length ABC fragment was able to activate the reporter in all three stem-cell-like cells, but not in differentiated teratoma-derived cells grown without selection (Fig. 4b). Surprisingly, region A, predicted to be the main regulatory region in stem cells, promotes a significant reporter expression only in F9 EC cells. Activation of the mmu-miR-302 host-gene in ES cells and teratoma derived stem cell like cells required the entire 2.1 kb upstream region. In contrast, but not unexpected, we found that the first 500 bp of the upstream region was sufficient to strongly repress the baseline expression in differentiated teratoma-derived cells, which had lost their morphological stem cell characteristics^25^.

For all stem cells, the expression driven from the fragments A and AB was lower than expression driven from the entire 2.1 kb ABC fragment, indicating a possible repressor function in this region.

## Discussion

The structure of the miR-302 host-gene is conserved between mice and humans. The main difference is the alternative splice donor site in the mouse gene while the human homolog contains an alternative second exon. Splicing and poly-adenylation have been shown to influence the transcriptional activity of genes^31–33^, and our data suggest differential splicing and polyadenylation in different cell types. We found more than 54% of the miR-302 host RNA in the cytoplasm. This profs the export of the miR-302 host-gene RNA from the nucleus, however, the proportion in the cytoplasm is low compared to that found for the protein coding mRNAs we used as reference RNA transcripts. This could either reflect a short half-life time of the miR-302 host-gene RNA or a low export rate from the nucleus. With no data on a possible function of this lncRNA, it is impossible to speculate if this export is of any functional relevance. In fact, the export might be a consequence of the functionally important processing of the miR-302 host-gene RNA. Capping, splicing and poly-adenylation are essential elements of transcriptional regulation^31,33^ and it is intriguing that the sequences responsible for the RNA processing are highly conserved compared to the actual sequence. While this is highly speculative, it would explain why the RNA is quickly degraded and removed from the cytoplasm.

We also found high conservation of TF binding sites in at least 2 kb of upstream genomic region, considerably more than the 500 bp so far annotated as promoter of the hsa-miR-302 host-gene^8,9,34^. The upstream genomic region also contains binding sites for tissue-specific transcription factors like GATA-6 or HNF-3ß and inhibitors like HBP1 and SP100. This might explain why *miR-302*s are also suggested as a marker for acute heart failure^35^.

*MiR-302*s are expressed in ES, EC and iPS cells^9,34,36,37^, but expression is elevated in primed compared to naive ES cells^30,38^, correlating with the lower level of expression we observe in ES cells compared to EC cells.

Using TRANSFAC, we identified potential binding sites for transcriptional repressors including HBP1 (at -825), SP100 (at -782), BCL-6 (at -419), HIC1 (at -269 and -848) and GFI1 (at -565) as factors unique for region AB (Fig. 4a). HBP1 is an inhibitor of the WNT signaling pathway^39^ that inhibits the cell cycle^40^, and can directly inhibit the DNA binding of the TCF4/β-catenin complex^39^. HIC1 is a transcriptional repressor and a tumor suppressor that negatively regulates the WNT signaling pathway^41,42^. GFI1, a zinc finger protein, is a transcriptional repressor that controls histone modification. It has a significant effect on self-renewal of hematopoietic stem cells and myeloid and lymphoid differentiation^43^. The proof of such possible repressive function would require ChIP-seq data, which to our knowledge are not yet available for these factors in mouse, or alternatively functional transcription assays, which are beyond the scope of this study.

Our analysis showed, that region AB has an ambiguous regulatory function, which might explain the cell type specific expression level of the mmu-miR-302 host-gene in otherwise very similar types of stem cells. It remains to be investigated, to which degree and in which cell types the possible TF binding sites actually are functional.

The *miR-302* cluster is embedded in the intron of its host-gene, which is transcribed as a lncRNA. This raises the general question why some miRNAs, like *miR-302*, have non-coding host-genes. Alternatively, they could be transcribed by RNA pol-III or be located in the introns of coding genes. Transcription from an RNA pol-III promoter does not offer the complex expression pattern observed for many microRNAs. For a microRNA cluster like *miR-302*, an evolutionarily easy way to recruit a pol-II promoter would be the localization in an intron of a gene encoding one of its major transcription factors (e.g., OCT4), which should give the appropriate regulation of expression. However, the complexity of the miR-302 host-gene expression exceeds that of any single transcription factor involved in its transcription, which explains why the *miR-302* cluster requires its own transcriptional regulation. Assuming that no existing gene recapitulates the expression required for *miR-302* expression, it seems, from an evolutionary point of view, easy to drive the expression from a non-coding gene, which will tolerate changes in transcription as long as no other function is coupled to it.

It is therefore conceivable that a major function of the miR-302 host-gene is to provide a regulatory transcription scaffold for the *miR-302* cluster. The absence of additional functions is obviously difficult to proof and we only have investigated one example. However, it might be worthwhile to add the transcriptional regulation of embedded miRNAs into the possible functions of 'orphan' lncRNAs.

## Supplementary Information

Supplementary Information
