# Supplementary material for: Expression of the miR-302/367 microRNA cluster is regulated by a conserved long non-coding host-gene
Source: Sci Rep. 2021 May 27;11:11115. doi: 10.1038/s41598-021-89080-z (PMC8159989; doi:10.1038/s41598-021-89080-z)
Supplement: Supplementary file 1 — Supplementary Information [file 41598_2021_89080_MOESM1_ESM.pdf]

Supplemental information:

Expression of the miR-302/367 microRNA cluster is regulated by a conserved long non-coding host-gene

Karim Rahimi[1,*], Annette Christine Füchtbauer[1], Fardin Fathi[2], Seyed Javad Mowla[3], Ernst-Martin Füchtbauer[1,*]

[1] Department of Molecular Biology and Genetics, Aarhus University, Denmark

[2]Cellular and Molecular Research Center, Research Institute for Health Development, Kurdistan University of Medical Sciences, Sanandaj, Iran.

[3] Molecular Genetics Department, Faculty of Biological Sciences, Tarbiat Modares University, Tehran, Iran

* Correspondence: Ernst-Martin Füchtbauer (emf@mbg.au.dk); Karim Rahimi (karim@mbg.au.dk)

Tel.: +45-28992238 (EMF)

Address: C.F. Møllers Alle 3, Aarhus C, Dk-8000, Denmark

List of primers and oligo sequences

Primers were designed using CLC main work bench (Qiagen) and checked by NCBI primer-BLAST and ordered from Sigma-Aldrich and used at a concentration of 1 µM for PCR..

Table S1: Primers sequences

**Name**	**5′-3′ sequence**	**bp**
mmu-miR-302 promoter fwd	AAGAATATTAATGTTTCCTGGTTGCTTCTAAT	32
mmu-miR-302promoter rev exon 1	AAGAATGCTAGCTGACCGCCTCCCAAAGAGTCCTGTTC	38
mmu-miR-302 transcript fwd1	CTCCGAGGACAGAACAGGA	19
mmu-miR-302 transcript fwd2	AGAACAGGACTCTTTGGGAG	20
mmu-miR-302 transcript fwd3	TCACGAAGGGTCCCACA	17
mmu-miR-302 transcript fwd4	CACGAAGGTAAAGGAGGGT	19
mmu-miR-302 transcript fwd5	GCTTTTGCTGCTTGCTCTTTT	21
mmu-miR-302 transcript fwd6	AACCACATTGCCACATTTCCCA	22
mmu-miR-302 transcript rev1	ATTTATAATTCATTTATTGG	20
mmu-miR-302 transcript rev2	TATTACAGGAGTTGCTC	17
mmu-miR-302 transcript rev3	GCTCCCCAAAAATGTTACTCA	21
mmu-miR-302 transcript rev4	GGATTTGCCTTTGTGGAA	22
adapter overhang primer	GCGAGCTCCGCGGCCGCGTTTTTTTTTTTT	30
anchor overhang primer	GGCCACGCGTCGACTAGTACTTTTTTTTTTTTTTTTT	37
mmu-miR-302pFwd1	AAGAATACCGGTCTGGAGTTGCTTTGTTTTC	31
mmu-miR-302pRev1&2	AAGAATCATGTTAAAGCAGAGGGGA	25
PCR primer 1 (PP1)	TCATACACATACGATTTAGGTGACACTATAGAGCGGCCGCCTGCAGGAAA	50
PCR primer 2 (PP2)	TAGACTTAGAAATTAATACGACTCACTATAGGCGCGCCACCG	42
HPRT +1	GCAAGCTTGCTGGTGAAA AGGA	22
HPRT -1	GCAGAUGGCCACAGGACUAGAACA	24
Malat1-fwd	TGAAGGTCGGCCTTGTAGAT	20
Malat1-rev	AACGGCCGTCAACTTAACCT	20
Gapdh-fwd	GGTGAAGGTCGGTGTGAACG	20
Gapdh-rev	CTGGCTCCTGGAAGATGGTG	20
Oct4-fwd	TCTTTCCACCAGGCCCCCGGCTC	23
Oct4-rev	TGCGGGCGGACATGGGGAGATCC	23

Programs used for PCRs

mmu-miR-302 host-gene promoter (short region A) PCR:

94 ˚C 5 min, (94 ˚C 1 min, 58 ˚C 45 sec, 72 ˚C 1 min) x 35, 72 ˚C 7 min

Forward primer: mmu-miR-302 promoter fwd1

Reverse primer: mmu-miR-302promoter rev exon1

mmu-miR-302 **spliced host RNA PCR:**

94 ˚C 5 min, (94 ˚C 1 min, 56 ˚C 1 min, 72 ˚C 1 min) x 35, 72 ˚C 7 min

Forward primer: mmu-miR-302 transcript fwd2

Reverse primer: mmu-miR-302 transcript rev3

Hprt **PCR:**

94 ˚C 5 min, (94 ˚C 45 sec, 60 ˚C 45 sec, 72 ˚C 1 min) x 30, 72 ˚C 7 min

Forward primer: HPRT +1

Reverse primer: HPRT -1

5' RACE PCR:

94 ˚C 5 min, (94 ˚C 1 min, 58 ˚C 1 min, 72 ˚C 1 min) x 35, 72 ˚C 7 min

Forward primer: PCR primer 1 (PP1)

Reverse primer: mmu-miR-302 transcript rev4

3' RACE PCR:

94 ˚C 5 min, (94 ˚C 1 min, 54 ˚C 1 min, 72 ˚C 1 min) x 35, 72 ˚C 7 min

Forward primer: mmu-miR-302 transcript fwd6

Reverse primer: PCR primer 2 (PP2)

Program for all qPCRs:

94 ˚C 10 min, (94 ˚C 30 sec, 56 ˚C 30 sec, 72 ˚C 30 sec) x 40, 95 ˚C 1 min, 55 ˚C 30 sec, 95 ˚C 30 sec

Table S2: Software and online tools used for analyzing of miR-302 host-gene

	**Software or online tools**
1	Ensembl, NCBI and UCSC genome browsers
2	miRBase (http://www.mirbase.org)
3	miRDB (http://mirdb.org/miRDB)
4	Rfam (http://rfam.xfam.org/)
5	promoter 2.0 prediction server (http://www.cbs.dtu.dk/services/Promoter)
6	Neural Network Promoter Prediction (http://www.fruitfly.org/seq_tools/promoter.html)
7	WebGene (http://www.itb.cnr.it/webgene)
8	Functional RNA Analysis (https://www.ncrna.org/)
9	GeneMark (http://exon.gatech.edu)
10	Transcriptional Regulatory Element Database (http://rulai.cshl.edu/cgi-bin/TRED/tred.cgi?process=home)
11	Mammalian Promoter Database (http://rulai.cshl.edu/cshlmpd/release.html)
12	Mouse Genome Informatics (http://www.informatics.jax.org)
13	CLC main work bench (https://www.qiagenbioinformatics.com/products/clc-main-workbench)
14	Transcription Factors Binding Sites tool, TRANSFAC (http://www.biobase-international.com/product/transcription-factor-binding-sites)
15	MaxEntScan (http://genes.mit.edu/burgelab/maxent/Xmaxentscan_scoreseq.html)
16	GeneSplicer (http://ccb.jhu.edu/software/genesplicer)

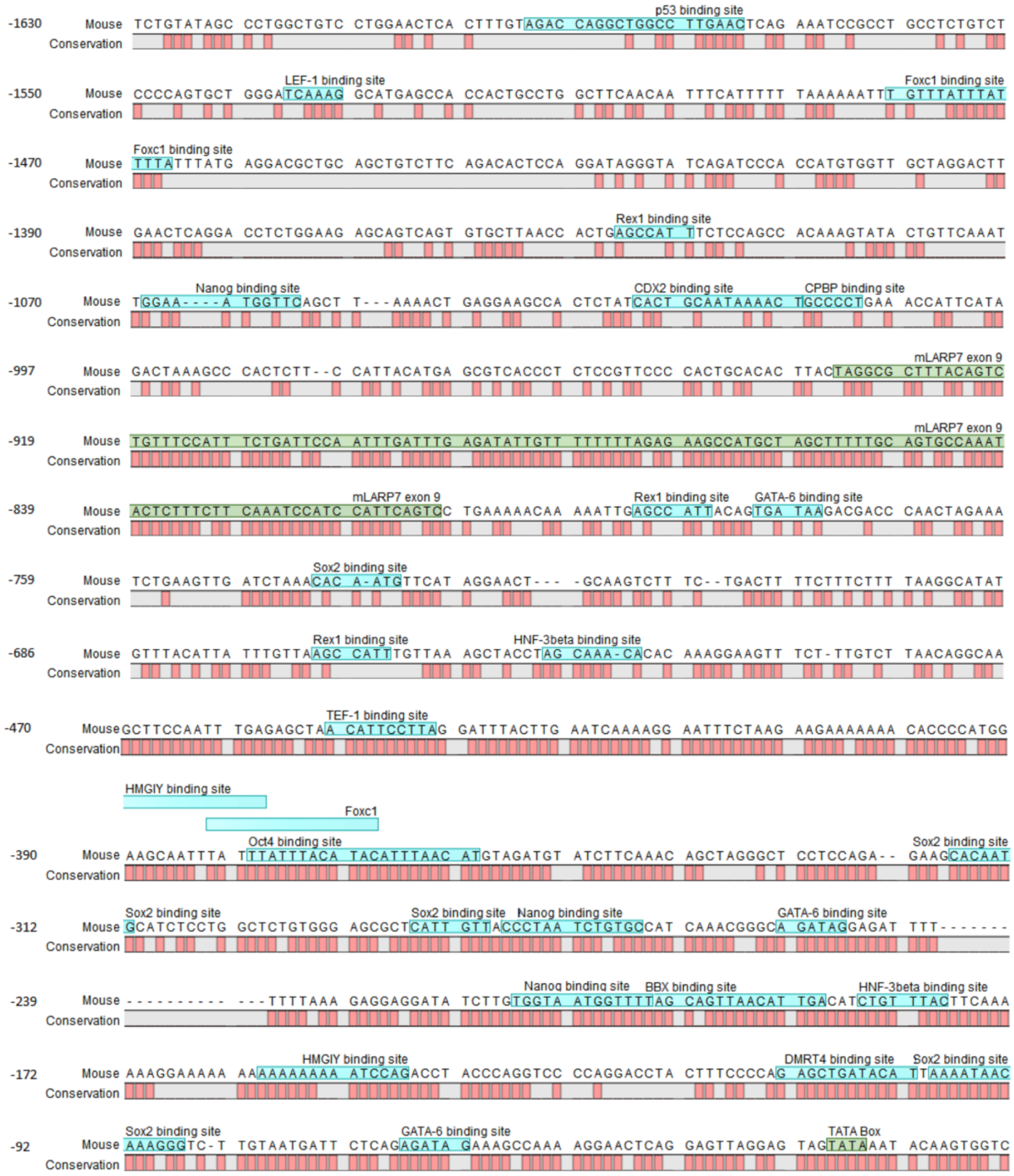

Figure S1. Conservation of the miR-302 host-gene promoter between mouse and human. The murine sequence shown is located at chr3:127,543,459-127,545,075 - GRCm38/mm10 plus strand (-14 bp to -1,630 bp). Some of the predicted TFs binding sites (blue overlay) and sequence conservation with the human gene (red bars) are annotated. OCT4, SOX2, and NANOG are the most important stem cell specific TFs that have binding sites in this region. Exon9 of *Larp7* in antisense direction and the miR-302 host-gene TATA-box are shown with a green overlay.

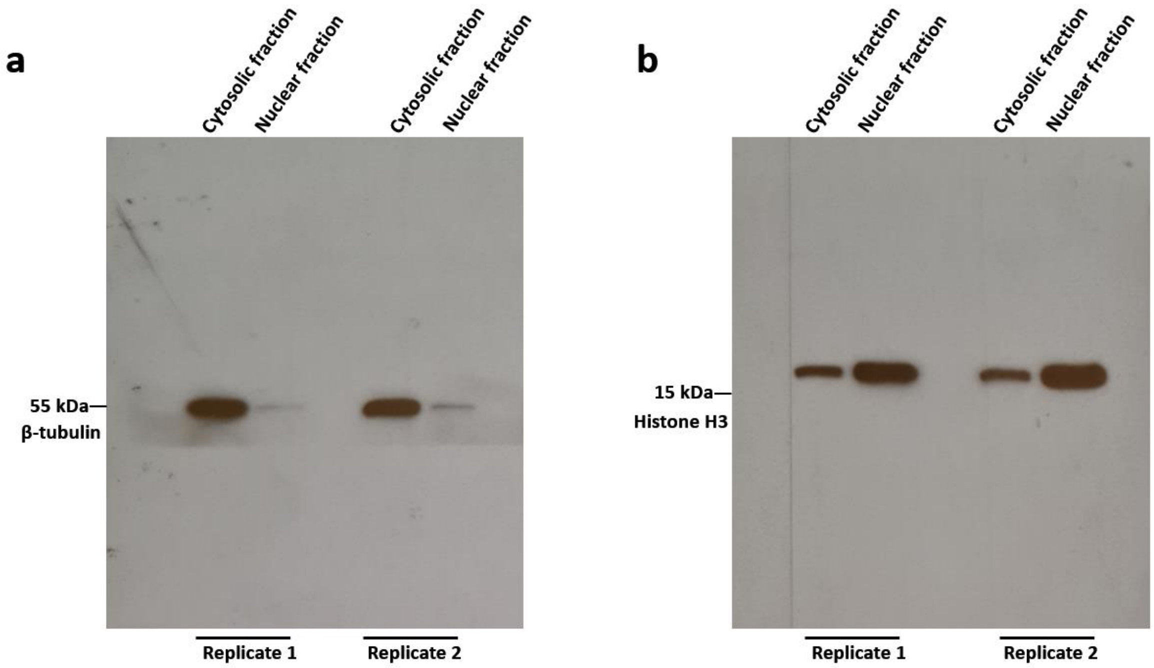

Figure S2. Full gel pictures of Western blot membranes hybridized with anti-β-tubulin (a) and anti-histone H3 (b) in both cytoplasmic and nuclear fractions and in two biological replicates. These fractions were used for investigation of miR-302 host RNA localization. The expected protein band sized for β-tubulin and histone H3 are ~55 kDa and 17 kDa respectively. The Western blot analysis confirm highly efficient fractionation. A small amount of β-tubulin in the nucleus is expected. Histone H3 is expected to be mainly in the nucleus while newly translated protein will be found in the cytoplasm before import to nucleus.
